# Leveraging Medico-Legal Interventions as a Last Resort to Save the Lives of High-Risk Mothers and Children: Case Studies From Rural Haryana, India

**DOI:** 10.7759/cureus.74496

**Published:** 2024-11-26

**Authors:** Ankit Chandra

**Affiliations:** 1 Centre for Community Medicine, All India Institute of Medical Sciences, New Delhi, New Delhi, IND

**Keywords:** healthcare access barriers, health services accessibility, maternal and child health, multi-agency collaboration, primary health care

## Abstract

Vulnerable groups, such as pregnant women and young children, can face barriers to timely and essential healthcare, primarily due to their dependence on caregivers. Medico-legal interventions are effective tools to protect high-risk populations when traditional methods fail. Based on my experience as a Medical Officer In-Charge of a Primary Health Center in rural Haryana, India, I present three case studies where legal assistance was used to ensure necessary medical care. The first case illustrates the role of culturally sensitive counseling and legal aid in overcoming resistance to neonatal care, while the second case highlights multi-agency support in addressing family instability and neglect. The third case demonstrates the efficacy of legal interventions to counteract familial reluctance in a maternal health crisis. These cases underscore the importance of incorporating legal support within healthcare frameworks to save the lives of high-risk mothers and children. Although medical professionals are traditionally taught to protect themselves from legal liability, the curriculum and training could also include guidance on using laws proactively to safeguard vulnerable patients in the community.

## Introduction

The maternal mortality ratio (MMR) in India for 2018-2020 was recorded at 97 per 100,000 live births [1], while the under-five mortality rate (U5MR) in 2020 stood at 32 per 1,000 live births [2]. The National Health Policy 2017 sets goals to reduce the MMR to below 100 per 100,000 live births and the U5MR to below 23 per 1,000 live births [3]. Although numerous interventions have been implemented, leading to a gradual decline in mortality rates, there remains an underutilized, effective approach: medico-legal interventions, which could play a crucial role in safeguarding vulnerable and underserved pregnant women and children [4,5].

Pregnant women and young children are susceptible to adverse health outcomes due to their dependence on caregivers for healthcare decision-making and access [6,7]. The timeliness and quality of care they receive often hinge on caregivers' perceptions, understanding, and willingness to seek medical help [8]. In some cases, caregivers' or family members' ignorance or reluctance can be life-threatening for patients [9]. Medico-legal interventions encompass actions at the intersection of medical practice and the legal system. Sometimes, legal measures are necessary to ensure that healthcare is delivered, and patients' rights are protected. These interventions are often utilized in public health policies and laws to protect population health, but they can also be applied on an individual level by healthcare practitioners to provide quality and essential care to neglected patients [10].

As the Medical Officer In-Charge of the Primary Health Center (PHC) in Chhainsa, rural Haryana, India, I have encountered situations where, despite extensive counseling and repeated efforts by our team, families refused or delayed essential care. When conventional methods fail, medico-legal support can be considered as a last resort to safeguard the health of vulnerable individuals. Through these examples, I aim to demonstrate that legal measures can be an effective tool in situations where conventional healthcare approaches encounter resistance or fail, and medico-legal assistance is essential to save the lives of at-risk mothers and children.

## Case presentation

About the field

PHC Chhainsa serves a population of approximately 52,000 through its six sub-health centers across eleven villages. The nearest sub-district hospital is located about 20 kilometers away, while the district hospital in Faridabad is 30 kilometers from PHC Chhainsa. Both PHC Chhainsa and the sub-district hospital, Ballabgarh, are part of the Comprehensive Rural Health Services Project in Ballabgarh, under the Centre for Community Medicine (CCM) at the All India Institute of Medical Sciences (AIIMS), New Delhi. Support and supervision for PHC Chhainsa are provided by the CCM, AIIMS, and the Government of Haryana.

Case study 1

Severe Neonatal Jaundice in a Preterm Infant and Familial Resistances

On May 17, 2024, during a home visit, an Auxiliary Nurse and Midwife (ANM) identified a seven-day-old female baby with severe jaundice in a remote village. Despite an hour of counseling, the family refused to take the baby to a hospital for evaluation. The ANM shared the photos and details with me over the phone; the baby was visibly jaundiced, and her condition appeared serious. The baby had been born prematurely (at 34-35 weeks gestation) with a birth weight of 2 kg at a private hospital, and although immunizations were recommended, none had been administered.

Over the phone, I explained the potential complications of severe jaundice to the family. The family agreed to seek care at a private hospital, according to their preference. However, later that night, the ANM informed me that the family had not followed through.

The next morning, the ANM and I visited the family’s home. Initially, they claimed the baby had been taken to Rajasthan for care. Sensing deception, as confirmed by neighbors, I attempted to connect with the family on a cultural level. I pointed to a religious photo in their house, invoking cultural teachings about the duty to protect children, especially female infants often seen as embodiments of goddesses. Reassuring the family, I explained that we were there to help. This approach seemed to resonate, and they eventually allowed us to see the baby, who was hidden inside the house. The baby’s condition had worsened-she appeared weak, and jaundice was evident in her palms and soles. I showed them a photo from the previous day to illustrate the progression of her condition.

The baby’s grandfather dismissed the severity, noting that their older grandson had also experienced jaundice and recovered with home care. I addressed their concerns rationally, asking what barriers were preventing them from seeking medical attention. They cited the distance to the district and sub-district hospitals, the lack of public transportation, and their previous experience being bad in the bigger hospitals. To overcome these obstacles, we offered them a free ambulance, assured them that we would make necessary arrangements for their comfort, and agreed to provide meals and lodging for the caregiver at the sub-district hospital. Although they were open to care at our PHC Chhainsa, they were reluctant to go to higher centers; unfortunately, the PHC Chhainsa lacked laboratory facilities to test bilirubin levels and phototherapy equipment. The family also mentioned financial constraints, although their living conditions appeared stable. Inquiring about the job profile, we learned that the baby’s father was a lab technician, which surprised us given the resistance to medical care. The grandfather of the baby runs a general store in the village. We then suggested a blood sample to assess bilirubin levels at a private lab to confirm the need for hospital care.

Seeing our dedication, the family finally agreed to visit the sub-district hospital. Before the family could change their decision, we didn’t wait for the ambulance; I took the mother, father, and baby with me in my personal car to the sub-district hospital. There, I prioritized her case, and the pediatrician recommended intravenous fluids, lab tests, and phototherapy after admission. The baby had lost 25% of her birth weight, and her total bilirubin level was 18.2 mg/dL. She was admitted overnight, and we arranged meals and accommodation for the father. However, the following morning, the family took leave against medical advice and returned home, ceasing further care.

**Figure 1:**
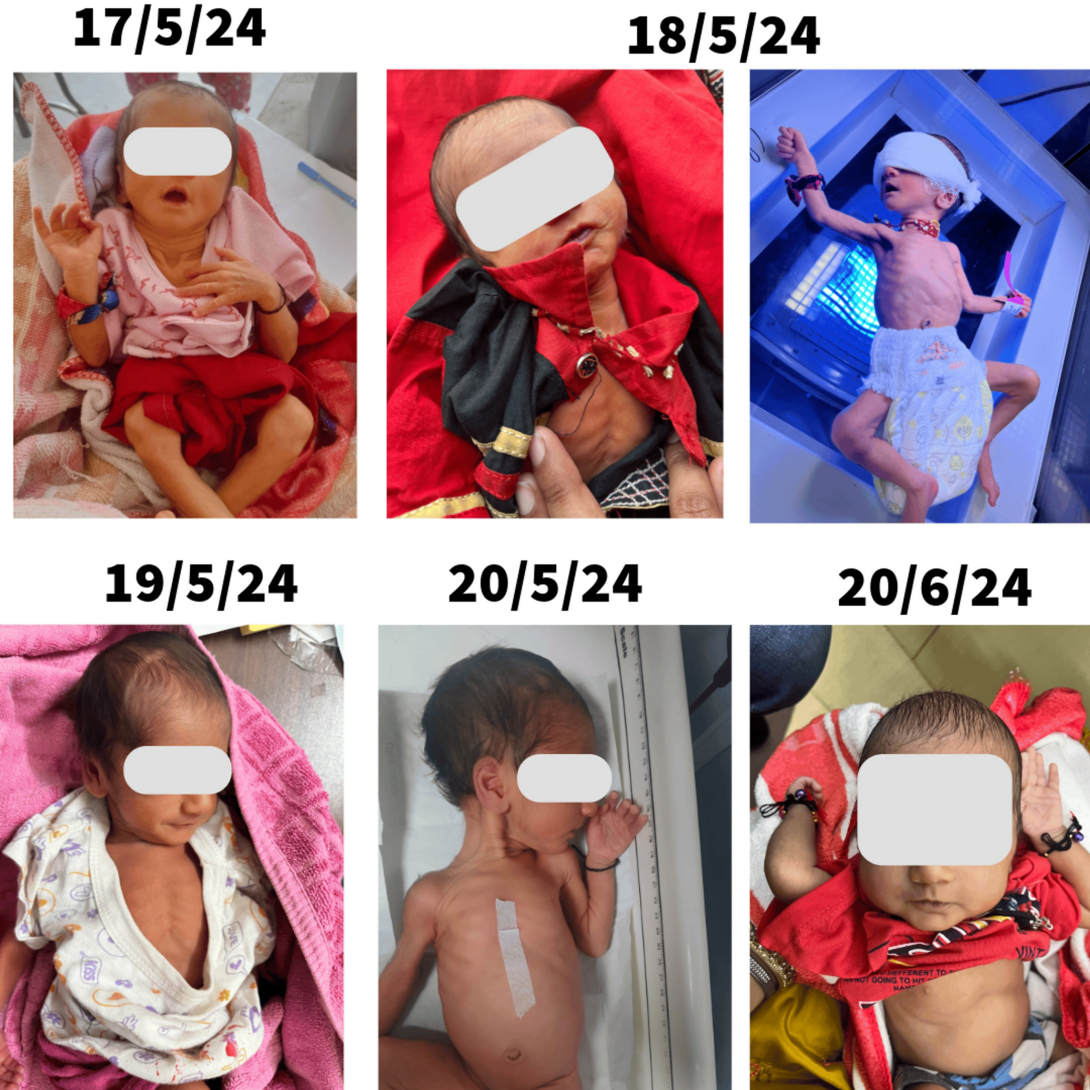
Timeline of clinical condition and interventions, showing the infant's physical appearance on consecutive days from May 17, 2024, to June 20, 2024. Written informed consent to include these images in the published article was obtained from the patient's legal guardian.

This act of neglect couldn’t be tolerated; we couldn’t just sit by and witness a neonatal death. This compelled me to call the child helpline and report the situation. A district child helpline member, accompanied by police and an ambulance, visited the family’s home. The entire village had gathered near their house upon witnessing the neglect of care for a female child. The villagers also urged the family to take her for the necessary treatment. Through a teleconsultation with a pediatrician, we secured an order for the baby’s readmission. Faced with legal consequences, the family complied, and the baby received necessary medical care at the sub-district hospital over the following days, ultimately recovering. Weeks later, she gained weight, and the family brought her to the sub-center for immunizations. It was a moment of joy for our team (Figure 1).

Case study 2

Neglect of Female Child and Family Instability

A three-month-old female child was brought to an immunization session at the subcentre by her mother. The child had diarrhea, and for further consultation, she was referred to me. On examination, the child presented with diarrhea with severe dehydration and acute malnutrition. While discussing the child’s condition, I learned that this was her mother’s third child. Her two-year-old sister was unable to stand or walk and was underweight, while her eldest brother, aged 5-6 years, appeared healthy.

The children’s father is an alcoholic with a history of domestic instability; his first wife left him, taking their children. Currently, the mother and her husband frequently argue due to infidelity and personal conflicts. The mother often takes her two daughters to her mother’s (maternal grandmother's) home for refuge. The maternal grandmother is a widow, lives alone, and works as a laborer. This child was fed packet milk through a bottle, as her mother was unable to breastfeed. The mother also uses a local chewable tobacco product and sometimes forgets to feed the child. This pregnancy was unplanned, and despite the mother's initial decision, the child’s paternal grandmother insisted against abortion, viewing it as a sin.

Given the child’s condition, I wanted her to be treated by a pediatrician at the sub-district hospital. However, recognizing the lack of family support, I decided to admit her to my PHC and started intravenous fluids. Since the family lives near the PHC, this would make it easier for them to comply with the admission. We also started intravenous antibiotics and symptomatic treatment, with plans for nutritional rehabilitation. On the second day, the child’s father, visibly intoxicated, came to the PHC and demanded that his wife return home. When she resisted, insisting on staying with her ill child, he callously said that the child could die at home if necessary. This escalated into a confrontation where the father fought with staff and forcibly took the child back home.

Concerned for the safety of the mother and her children, I contacted the child helpline, which advised us to call the police for the immediate rescue of the child, as their team would take time to arrive. Accompanied by the police, the ANM and I visited the family's home. We found the children on a bed, the father intoxicated, and the mother sweeping the house. She appeared relieved upon seeing us and asked for help. The police took her statement, and we brought her, along with her two daughters, back to the PHC, where we resumed the treatment for the sick child.

In the urgency of the situation, we had forgotten to gather their clothes and essential documents. When we sent a friend of the mother to collect their belongings, the father refused access, demanding a complete separation from his wife and issuing threats of suicide and harm. That night, a relative of the father arrived at the PHC, brandishing a stick and verbally abusing the mother. Villagers gathered to watch, but no one intervened. Our security guards prevented the relative from entering, ensuring safety. The mother then called the police for protection and reached out to the maternal grandmother of the child. By the time the police arrived, the crowd had dispersed. We provided the mother and her daughters with food and basic necessities during their stay at the PHC.

**Figure 2:**
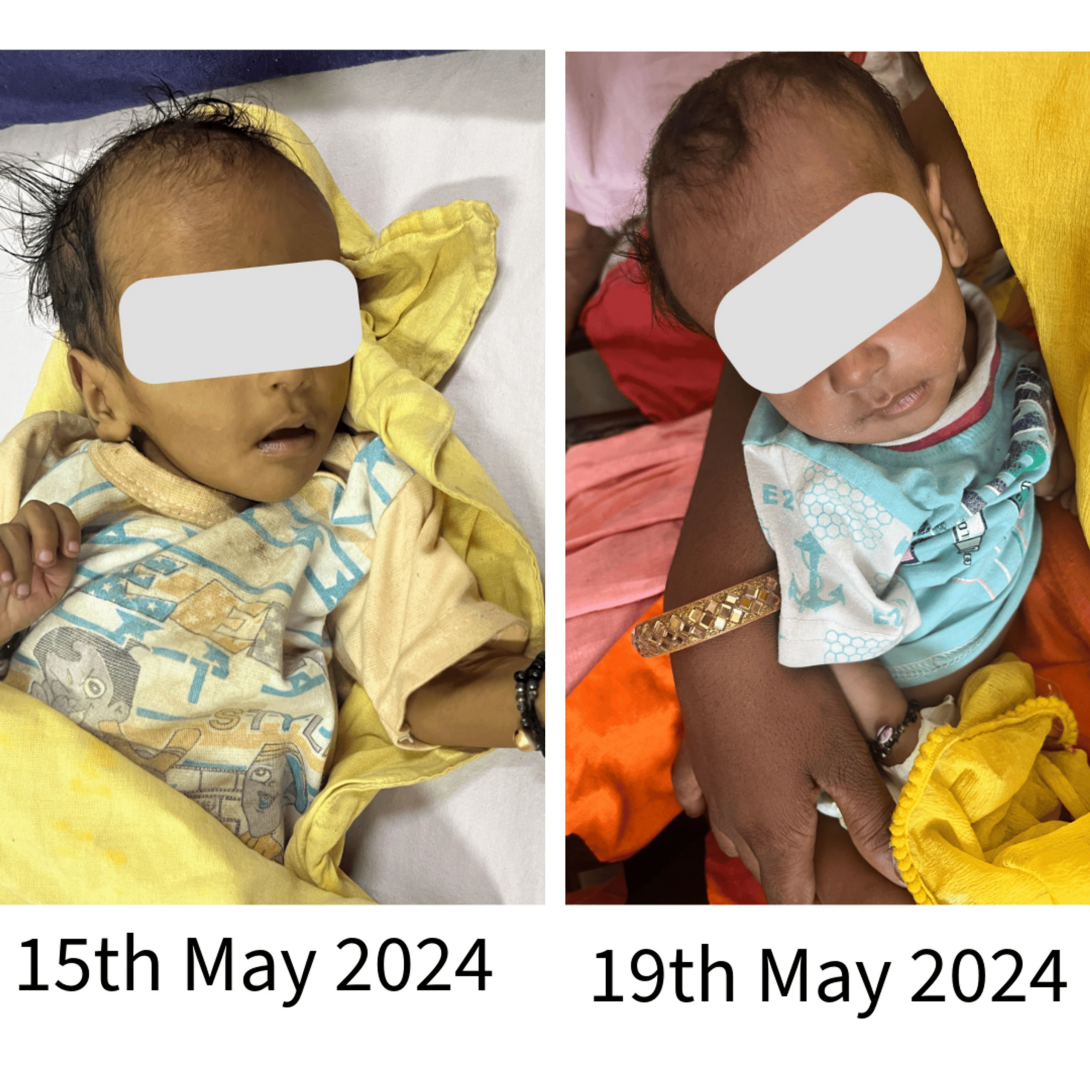
Images depict the physical condition of the three-month-old female child on May 15, 2024, and show diarrhea with severe dehydration and acute malnutrition on May 19, 2024, after initial interventions at the Primary Health Center. Written informed consent to include these images in the published article was obtained from the patient's legal guardian.

The next morning, the maternal grandmother of the child arrived at the PHC to support her daughter and granddaughters. The child welfare team was informed of the situation; they planned to come at discharge to address the family conflict. After four days of treatment, the child improved and was shifted to oral medications (Figure 2).

The child welfare team visited the PHC with the police. They spoke with the mother and called her husband and her mother-in-law. The paternal grandmother brought the necessary documents and clothing. Due to safety concerns, a mutual agreement was reached: the mother and her two daughters would reside at the maternal grandmother’s house, while the child welfare team would conduct follow-ups to ensure their wellbeing. The mother was reassured of the availability of social support schemes and potential sponsorship for her and her children.

After a few weeks, we received an update from the ANM that the mother and her daughters were doing well at the maternal grandmother’s house. She has quit tobacco and started daily work to support and feed her children.

Case study 3

Severe Anemia in a Pregnant Woman and Family Conflict

On July 10, 2024, the ANM from one of our subcentres informed me about a pregnant woman, G4P2L1A1D1, with a period of gestation of 27+6 weeks attending the antenatal clinic, who had a hemoglobin level of 3.9 gm/dL. She was referred for a blood transfusion at a district hospital. In June 2024, this patient received three doses of intravenous iron sucrose 300mg each at PHC Chhainsa with a hemoglobin of 6.8 mg/dL, but did not respond well.

On 11th July, she again presented to the subcentre with a hemoglobin level of 3 gm/dL, complaining of fatigue and palpitations. This time, her family took her to the district hospital, where she was found to have pedal edema and breathlessness, with a hemoglobin level measuring 2 gm/dL. Due to the unavailability of blood or potential complications, she was referred to a medical college in New Delhi, located half an hour away from the district hospital. However, her husband claimed he didn’t have time to travel to Delhi and brought her back home. The following day (12th July), our Accredited Social Health Activist (ASHA) and ANM visited the house to counsel the family on the importance of going to a higher center to save both her life and that of her baby. Despite multiple visits throughout the day, the family remained unresponsive to the counsel, repeatedly delaying the hospital visit.

Upon further inquiry, we found that her husband was a daily wage worker who had recently purchased a motorcycle with some savings, which had caused conflict with his mother. The mother-in-law was upset that her son was spending money on non-essential items.

**Figure 3:**
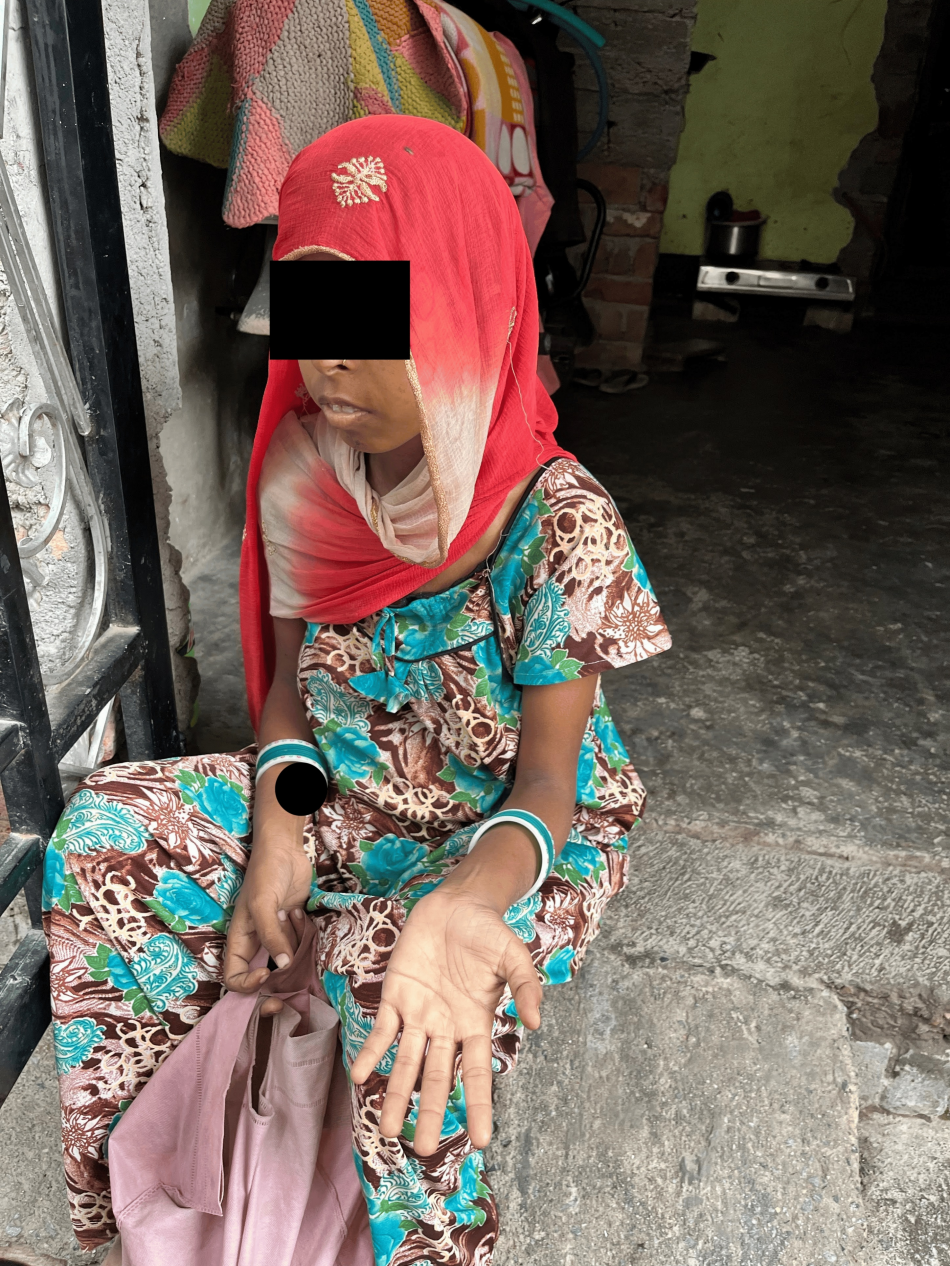
The image shows the patient with visibly pale palms and lips, indicating severe anemia, while a newly purchased motorcycle is visible in the background. Written informed consent to include this image in the published article was obtained from the patient.

On 13th July, I visited their home with our health workers. The family lived in a concrete house on a muddy road filled with filth. A new motorcycle was parked inside, while houseflies were everywhere. The mother-in-law was washing utensils outside. When asked why they hadn't taken the patient for a blood transfusion, she responded that it was the husband's responsibility to care for his wife, not just spend money on a bike. I saw the patient sitting in a corner, extremely pale, and visibly tired (Figure 3). On examination, she had bilateral pedal edema, mild crepitations in her lungs, and tachycardia.

Realizing that family conflicts were putting both mother and child in grave danger, I called the husband and explained the critical nature of her condition and the consequences of not treating her severe anemia. The husband said he couldn't take his wife anywhere because he would lose his daily wages. I offered to arrange free transport, arranging the blood transfusion at the hospital and other essentials but emphasized that a relative or neighbor needed to accompany her to the hospital for an urgent transfusion. The husband was very reluctant and stated that they would not take her to any hospital. Instead, they planned to improve her diet, as it was currently poor, and they were prepared to face the consequences of their inaction.

As we were running out of time, and recognizing the gravity of the situation along with the family’s disregard for her life, I reached out to the National Women’s Helpline for some sort of help. I was determined to prevent maternal mortality in this scenario. The women's helpline told me that this was the first time they had received such a call. I explained to them that a woman’s life was in danger due to her medical condition and the ignorance of her family members, along with the potential legal implications. They sent the police to assess the situation and assist us, as we had planned to transfer her to Delhi for a blood transfusion. When the police arrived, the entire village gathered outside the house. Everyone in the village persuaded the mother-in-law for a transfusion, which caused some embarrassment and prompted her to consider the patient's well-being. Eventually, the mother-in-law called her son and agreed to the blood transfusion. Among the crowd, a distant female relative of the family, who was a native of the village, volunteered to accompany her to the hospital in Delhi.

Simultaneously, I spoke with my seniors and colleagues at the sub-district hospital to arrange a bed and necessary care at AIIMS New Delhi for this patient. We arranged for an ambulance, and one of our medical interns accompanied the patient to AIIMS Delhi and assisted with her admission. She received transfusions of 4 units of packed red blood cells and 8 units of platelets. The workup revealed megaloblastic anemia and pancytopenia. She was admitted and treated for a week at AIIMS Delhi by the Department of Obstetrics and Gynecology. Her husband and mother-in-law stayed with her during her hospital stay.

After her discharge, she followed up monthly at AIIMS Delhi and at our sub-center. She recovered well and was doing fine. After a few months, she delivered a healthy-term baby without any complications.

## Discussion

The first case underscores the role of culturally sensitive counseling and legal aid in overcoming familial resistance to medical intervention. In the second case, the father’s antagonistic behavior posed a barrier to healthcare. By involving the National Child Helpline and local police, the healthcare team facilitated intervention, ensuring the safety and well-being of the mother and children. This case demonstrates the importance of multi-agency support in protecting vulnerable family members and ensuring continuity of care. The third case highlights the potential for legal support to counteract familial reluctance in seeking healthcare. The legal intervention not only facilitated immediate care for the patient but also served as a community demonstration of the healthcare team’s commitment to patient welfare. By involving the police, the healthcare team communicated the seriousness of maternal health issues and fostered an environment of accountability.

My experiences in these cases were largely positive. Initially, I felt some hesitation about seeking legal assistance, but my conscience urged me not to ignore these patients. Legal assistance is available; however, it is often underutilized, and we aren’t trained in how to leverage it effectively in difficult situations in the field. In medical education, medico-legal aspects of case management are typically taught to protect doctors from lawsuits and complaints [11,12]. There is a need, however, to also apply these laws proactively to protect and save the lives of vulnerable patients in the community. Personal effort and perseverance are crucial to making a difference. While this approach may occasionally place us in difficult situations, the satisfaction of seeing positive outcomes is profoundly rewarding.

We often work in a particular area for extended periods and may avoid taking actions that could disrupt our relationship with the community. However, is it worthwhile to prioritize our comfort over the health and lives of others? Reflecting on my experience, I realized that, despite my initial concerns about potential community backlash, we received a positive response. In cases one and three, community members gathered to support the caregivers in taking timely action and even volunteered to accompany the patients during transportation. Community involvement fosters long-term support, as community members can continue to monitor and assist families with health needs, reinforcing trust and respect for the healthcare system. Community members appreciated the personal efforts I took on their behalf, seeing it as a sign of dedication and compassion. This experience boosted the confidence and morale of our health workers and earned us respect within the community. The local police and the personnel from the Women and Child Helpline were also highly supportive and enthusiastic. Involving them as an independent third party is crucial, as it shifts the issue beyond a personal matter, providing an objective and broader perspective. Additionally, their involvement helps protect the health team from potential repercussions.

One could argue that involving third parties or taking legal help could compromise a patient’s autonomy and breach confidentiality. However, when a life is at risk, it becomes essential to prioritize safety by involving legal and protective authorities. Communication with a helpline or the police may be justified as an exception to privileged confidentiality, as safeguarding life takes precedence. Furthermore, if local community groups, such as the gram panchayat, gram sabha, village forest committee, paani samiti, or Village Health, Sanitation, and Nutrition Committee are active in a village, they can also be engaged as a support system.

Medico-legal interventions should not be underestimated, as they have substantial potential to reduce maternal and neonatal mortality. Research has shown that legal empowerment can improve access to quality healthcare services, in part through community awareness [13]. However, it is equally important to raise awareness among medical practitioners, encouraging them to assist vulnerable people in need. Additionally, we must focus on enhancing women’s autonomy to improve healthcare utilization, achievable through women’s education and empowerment [14].

## Conclusions

Medico-legal support can be a valuable tool for healthcare providers in rural India to ensure care for dependent and vulnerable populations. These case studies and personal experiences from a primary health center show that leveraging legal frameworks can encourage compliance with life-saving medical recommendations and interventions and can also improve the community's perception of the healthcare system. Healthcare providers are encouraged to consider legal support as a viable last resort when conventional methods fail to save the lives of high-risk mothers and children.
